# Precision-Oriented Reconstruction After Spinal Sarcoma Resection: Integrating Surgical Strategy, Biologic Risk, and Emerging Technologies

**DOI:** 10.3390/cancers18101555

**Published:** 2026-05-11

**Authors:** Tanner Carcione, Bradley Callas, Jack Thiara, Walter N. Jungbauer, Jonathan Jeger, Edward Reece

**Affiliations:** 1 Division of Plastic and Reconstructive Surgery, Mayo Clinic, Phoenix, AZ 85054, USA; jungbauer.walter@mayo.edu (W.N.J.); jeger.jonathan@mayo.edu (J.J.); 2 School of Medicine, Creighton University, Phoenix, AZ 85013, USA; bradleycallas@creighton.edu (B.C.); jackthiara@creighton.edu (J.T.); 3 Division of Plastic and Reconstructive Surgery, Cleveland Clinic, Cleveland, OH 44195, USA

**Keywords:** spinal sarcoma, precision oncology, vascularized bone graft, spinoplastic reconstruction, biologic fusion, 3D-printed implant, hybrid construct, reconstructive algorithm, irradiated spine

## Abstract

**Background/Objectives**: Primary spinal sarcomas, encompassing both bone and soft tissue histotypes, demand individualized reconstruction due to heterogeneous tumor biology, anatomic complexity, and host environments compromised by radiation, systemic therapy, or prior surgery. This narrative review reframes post-resection spinal reconstruction through a precision-medicine lens. **Methods**: A structured literature review was performed using PubMed and Scopus, targeting articles published between 2000 and 2026. Searches encompassed spinal sarcoma reconstruction, radiation and fusion, biologic reconstruction, and emerging technologies. **Results**: Tumor grade, radiation exposure, and systemic therapy timing emerge as multiplicative determinants of reconstructive environment quality, with drug-class-specific perioperative effects warranting stratified management. Vascularized bone grafts achieve reliable fusion in compromised hosts where avascular constructs fail. A precision-oriented reconstructive ladder is proposed as a conceptual, hypothesis-generating framework to guide strategy selection. Hybrid PSI-VBG constructs may further expand reconstructive possibilities. The evidence base remains largely composed of small, retrospective series. **Conclusions**: Individualized strategies anchored in tumor biology and host environment are the cornerstone of durable spinal sarcoma reconstructions. The proposed framework requires prospective, multi-institutional validation. Standardized outcome definitions, prospective registries, and histotype-stratified analyses are needed to advance the field.

## Simple Summary

Primary spinal sarcomas are rare tumors that require complex reconstruction after surgical removal. Current reconstructive approaches often use a uniform strategy regardless of tumor type, radiation history, or the patient’s expected survival, leading to high rates of complications such as failed bone healing and hardware failure. This review examines how tumor biology, radiation exposure, systemic therapy, and host tissue quality interact to determine reconstructive success or failure. We synthesize the available evidence on vascularized bone grafts, metallic implants, and emerging hybrid constructs, and propose a precision-oriented reconstructive ladder that matches strategy to the individual patient’s biologic and clinical context. The framework is intended to guide multidisciplinary surgical planning rather than serve as a validated algorithm. Prospective registries and standardized outcome measures are needed to advance evidence-based reconstruction in this challenging population.

## 1. Introduction

Primary sarcomas of the spine are rare neoplasms, representing fewer than 5% of all primary sarcomas, yet their clinical significance is disproportionate to their incidence [1]. These tumors encompass both soft tissue and primary osseous histotypes, including chordoma, chondrosarcoma, osteosarcoma, and Ewing sarcoma. Although their oncologic biology differs substantially, they share common reconstructive challenges: large post-resection defects, host environments compromised by radiation and systemic therapy, complex biomechanical demands, and extended survival horizons requiring durable constructs [2,3]. This shared reconstructive burden justifies their collective consideration in the present review, consistent with the approach taken by the WHO Classification of Tumours, which addresses bone and soft tissue sarcomas in a single combined volume [4], by the NCCN and ESMO guidelines, which group these histotypes under unified management frameworks [5,6,7], and by recent clinical registries that analyze bone and soft tissue spinal sarcomas as a unified cohort [8]. The Weinstein–Boriani–Biagini (WBB) surgical staging system, the standard framework for primary spinal tumor surgical planning, is itself histotype-agnostic and applies uniformly across all primary spine tumor types [9]. Unlike sarcomas of the extremities, where limb-salvage algorithms are well established and compartmental anatomy permits relatively straightforward surgical planning, spinal sarcomas demand a level of individualization that impacts every aspect of management [2,3].

Spinal sarcoma reconstruction is challenging for a variety of reasons outside of operative complexity. It is the mismatch between the uniformity of conventional reconstructive solutions and the substantial heterogeneity of the environments in which they must perform. A titanium cage placed in an unirradiated, well-vascularized bed in a patient with localized low-grade chondrosarcoma faces fundamentally different demands than one placed across a previously irradiated sacral defect in a patient with recurrent Ewing sarcoma who has undergone multiple prior operations.

**Figure 1:**
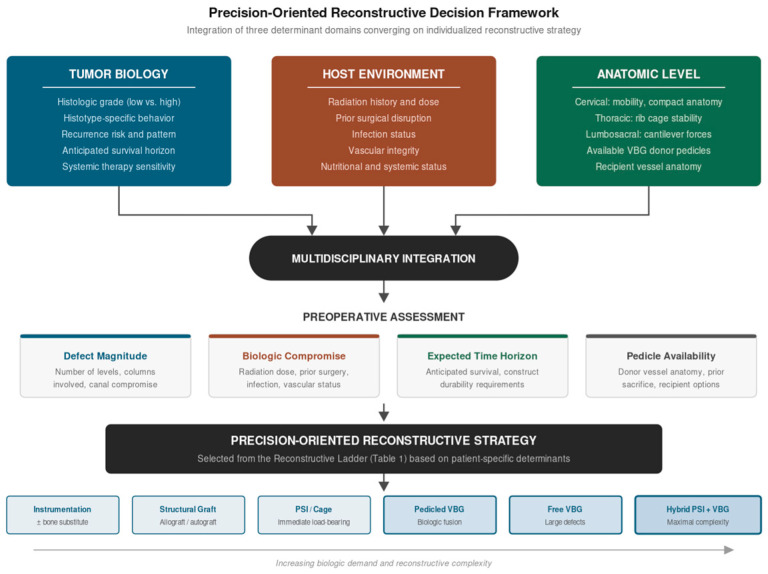
Precision-Oriented Reconstructive Decision Framework. Tumor biology, host environment, and anatomic level converge through multidisciplinary integration to determine individualized reconstructive strategy. Preoperative assessment of defect magnitude, biologic compromise, expected time horizon, and vascular pedicle availability guides selection from the reconstructive ladder.

This review proceeds from that observation. Rather than cataloging reconstructive techniques, we synthesize the available evidence to identify the biologic, oncologic, and host-level factors that should drive individualized reconstructive decision-making. For these complex cases, we propose a precision-oriented reconstructive ladder which integrates emerging technologies and novel surgical techniques. We identify the critical gaps in evidence where prospective investigation is urgently needed (Figure 1). The aim of this article is to better inform multidisciplinary management. Spinal sarcoma management demands extensive collaboration from oncologists, neurosurgeons, and reconstructive surgeons; this article should not serve as a validated algorithm, but rather a conceptual treatment paradigm that considers cellular, anatomic, and mechanical elements in providing an optimal reconstructive outcome.

## 2. Methods

A structured narrative literature review was performed using PubMed and Scopus databases. The primary search targeted articles published between 2000 and 2026, with emphasis on publications from 2020 onward. Earlier foundational and highly cited publications were included where they provided essential context. Search queries were organized into four thematic groups using Boolean operators: (A) (spinal sarcoma OR spine sarcoma OR vertebral sarcoma OR sacral sarcoma) AND (reconstruction OR reconstructive), (B) (spinal sarcoma) AND (radiation OR irradiation) AND (fusion OR healing OR pseudarthrosis), (C) (spinal sarcoma) AND (vascularized bone graft OR free fibula OR pedicled graft OR autograft OR biologic reconstruction), and (D) (spinal sarcoma OR spine tumor) AND (3D-printed implant OR patient-specific implant OR additive manufacturing OR navigation OR CTA). Targeted manual searches were performed for emerging technologies not well indexed in standard databases, and supplementary searches were conducted for systematic reviews, meta-analyses, and clinical practice guidelines across all thematic groups.

Both primary bone sarcomas (chordoma, chondrosarcoma, osteosarcoma, Ewing sarcoma) and soft tissue sarcomas of the spine were included, reflecting the shared reconstructive challenges these histotypes present and the approach taken by current WHO, NCCN, and ESMO classification and guideline frameworks [4,5,6,7]. Studies were included if they reported on primary spinal sarcoma, reconstruction after tumor resection, outcomes including fusion, durability, or complications, perioperative management relevant to reconstruction, or biologic and hybrid construct strategies. Studies focused exclusively on metastatic spine disease, degenerative spine fusion, non-oncologic deformity, or isolated case reports without clear novelty were excluded. Reference lists of included studies and relevant review articles were hand-searched to identify additional sources. Initial searches yielded 487 records; after removal of duplicates and screening by title and abstract, 142 articles were assessed for full-text eligibility, with 90 studies included in the final synthesis. Given the rarity of primary spinal sarcomas and the predominance of small, retrospective series, no formal meta-analytic synthesis was attempted; instead, findings are presented as a narrative synthesis with explicit attention to evidence quality and study limitations.

## 3. Biologic and Treatment Determinants of Reconstruction

The following sections present the findings of our structured literature review, organized thematically by the determinants of reconstructive decision-making. Because the evidence base for spinal sarcoma reconstruction is comprised predominantly of small, retrospective case series (Level IV evidence), the strength of any comparative claims is limited accordingly, and findings should be interpreted as hypothesis-generating rather than definitive.

### 3.1. Influence of Tumor Biology

Tumor biology is a primary determining factor in the surgical management, and ultimately reconstruction, of primary spinal sarcomas. The histologic grade, intrinsic aggressiveness, and recurrence risk not only determine surgical approach and margins but also dictate the extent, durability, and overall philosophy of reconstructive planning. In spinal sarcomas, reconstructive planning must account for the patient’s anticipated survival, risk of local recurrence, and the likelihood of future adjuvant therapy [10,11].

For low-grade spinal sarcoma, characterized by slower growth, lower metastatic potential, and favorable progression-free survival, patients should be managed as long-term survivors with emphasis on highly durable structural reconstruction [5,12]. Surgical management ideally includes en bloc resection with wide or marginal margins when feasible, with margins greater than 2 mm providing excellent local control and margins of 0.1 to 1.9 mm achieving reasonable outcomes [13]. The reconstructive implication is significant: low-grade tumors that are likely to be managed with surgery alone, without radiation, present the most favorable host environment for reconstruction. Longevity of reconstruction remains a principal concern in this patient population, as these patients are expected to live longest.

For high-grade spinal sarcoma, which demonstrate increased local aggressiveness, higher rates of local recurrence, and greater likelihood of systemic dissemination, en bloc resection remains the ideal surgical approach. Resection of high-grade spinal sarcoma is still potentially curative, although larger resections are usually required to achieve negative margins. Even still, there is a higher likelihood of adjuvant radiation or systemic therapy [14]. This creates a compounding challenge: high-grade tumors demand the most aggressive resection, generating the largest defects, while simultaneously requiring the adjuvant treatments that most compromise the wound bed upon which reconstruction depends. Tumor grade, resection magnitude, and treatment-related host compromise ultimately determine the survival of the reconstructive environment, and therefore must be anticipated prospectively rather than managed reactively.

Specific histotypes warrant individual consideration. Chordomas, despite their deceptively low histologic grade, are locally aggressive with local recurrence rates approaching 68% for cranial chordomas, with similarly high recurrence across spinal and sacral sites, and their resistance to conventional chemotherapy and radiation makes operative resection the cornerstone of management [15,16]. The reconstructive demands following chordoma resection are accordingly extreme, resulting in large spinal canal defects, frequently in previously irradiated fields after proton beam or high-dose photon therapy. Chondrosarcomas exhibit a wide behavioral spectrum from indolent low-grade lesions to dedifferentiated variants with aggressive metastatic potential: their resistance to both chemotherapy and conventional radiation doses makes the extent of surgical resection the primary determinant of local control [17,18]. Ewing sarcoma’s chemosensitivity and radiosensitivity distinguish it meaningfully from other subtypes, but the same neoadjuvant radiation that facilitates local control substantially compromises the host biology upon which reconstruction relies [19]. Spinal osteosarcoma, though rare, carries substantially worse prognosis than appendicular disease and commonly presents with extracompartmental extension requiring large, destabilizing resections in patients who are often young, making construct durability over decades a central planning concern [20].

### 3.2. Considerations for Radiation Therapy

Radiation therapy is frequently indicated in the management of primary spinal sarcoma to improve local control, particularly in high-grade tumors or when wide margins are not achievable due to anatomical constraints [21]. From a reconstructive perspective, radiation therapy is a significant modifier of wound biology, fusion capacity, and the vascular integrity of the operative field [22,23]. As a result, the timing of radiation therapy, cumulative dose effects, and anticipated changes in local vascularity should be considered during surgical and reconstructive planning [24].

The timing of radiation has important implications for wound healing and closure strategy. Neoadjuvant radiotherapy exposes tissues to microvascular injury and early fibrosis prior to surgery, increasing the risk of postoperative wound complications and reducing tissue tolerance for tensioned closures [24,25,26]. In these cases, reconstructive planning should emphasize robust soft tissue coverage and effective dead-space management [27]. In contrast, adjuvant radiotherapy may reduce early wound complications but exposes the healing incision and developing fusion mass to later fibrosis and vascular compromise, increasing the risk of delayed wound breakdown, hardware exposure, and impaired fusion [28,29]. The randomized trial by O’Sullivan et al. comparing neoadjuvant versus adjuvant radiotherapy in extremity soft tissue sarcoma demonstrated a higher rate of acute wound complications with neoadjuvant RT but lower rates of late fibrosis and joint stiffness, a tradeoff with direct relevance to spinal sarcoma sequencing decisions, where late fibrosis and hardware failure are dominant long-term concerns [24].

Radiation also exerts dose-dependent effects on soft tissue healing and arthrodesis. Endothelial injury, reduced angiogenesis, and impaired osteoblast activity contribute to delayed healing and an increased risk of pseudarthrosis [25,30,31]. At the doses commonly employed in spinal sarcoma, typically 50 to 66 Gy for definitive or adjuvant intent and higher doses in proton beam protocols for chordoma and chondrosarcoma, the probability of successful avascular graft incorporation in the treatment field is substantially reduced [22,23]. Reconstructive strategies in patients expected to receive radiation should therefore emphasize rigid fixation, robust structural integrity, careful preservation of tissue vascularity, and where the host environment is significantly compromised, prioritization of vascularized reconstruction. The reconstructive team should be engaged at the time of radiation so that field design and dose can be coordinated with the anticipated reconstructive approach, and donor vessel anatomy can be excluded from the high-dose field where oncologically feasible [32].

### 3.3. Effects of Systemic Therapy

Systemic therapy timing, including cytotoxic chemotherapy, targeted therapy, and immunotherapy, can influence wound healing, reconstruction durability, and treatment sequencing in patients undergoing oncologic spinal resection [33,34]. These effects are often underweighted in reconstructive planning discussions and warrant explicit preoperative consideration.

Cytotoxic chemotherapy impairs wound healing through inhibition of cellular proliferation, angiogenesis, and immune function, increasing the risk of postoperative wound complications if surgery is performed during treatment-related cytopenia. The nadir of myelosuppression typically occurs 7 to 14 days after administration, and most protocols recommend surgical timing to coincide with hematologic recovery, targeting an absolute neutrophil count greater than 1500 and platelet count greater than 100,000 [35,36]. However, the relationship between neoadjuvant chemotherapy timing and surgical site complications may be less straightforward than previously assumed. A secondary analysis of the PARITY multicenter randomized trial involving 216 sarcoma patients (75% osteosarcoma, 16% Ewing sarcoma) found that the interval from last chemotherapy cycle to surgery, with a median of 24 days, did not significantly impact surgical site infection or reoperation rates; longer operative time was the only independent predictor of complications [37]. While this finding pertains to extremity reconstruction rather than spinal surgery, it suggests that cytotoxic chemotherapy timing effects may be more modest than those of targeted agents. Preoperative assessment of albumin and prealbumin provides objective data to guide nutritional optimization, with intervention warranted in deficient patients before elective reconstruction [35].

Targeted therapies that inhibit angiogenesis carry a clearer and more substantial mechanistic association with wound-healing complications because VEGF signaling is essential for neovascularization during tissue repair. Drug-class-specific perioperative holding periods are recommended based on pharmacokinetic half-lives [36,38]. For bevacizumab (half-life approximately 20 days), a minimum 6-to-8-week preoperative washout is recommended for major surgery; a meta-analysis by Zhang et al. confirmed significantly increased wound-healing complications with perioperative bevacizumab [39]. For multi-targeted tyrosine kinase inhibitors such as pazopanib (half-life approximately 31 h) and sunitinib (half-life approximately 40 to 60 h), cessation of at least 5 half-lives, generally 1 to 2 weeks, is advised before surgery, with resumption deferred until wound healing is satisfactory [38]. A report from the Children’s Oncology Group ARST1321 trial provides the strongest prospective sarcoma-specific data on antiangiogenic wound effects: pazopanib combined with radiotherapy produced a 50% wound complication rate (47% grade III) compared with 22% in the chemotherapy-plus-radiotherapy arm, with pazopanib held 7 days preoperatively and 14 days postoperatively [40]. A comprehensive review by Okay et al. consolidates perioperative cessation protocols for cytotoxic, targeted, and immunotherapeutic agents relevant to orthopaedic oncologic surgery [36].

Checkpoint inhibitors (pembrolizumab, nivolumab; half-lives 12 to 25 days) appear broadly feasible in the perioperative setting, with most centers recommending a 3-to-6-week hold from the last infusion before major surgery and resumption 2 to 4 weeks postoperatively [36]. However, emerging evidence suggests that immune-modulating therapies may influence inflammatory and repair pathways involved in wound healing, with implications for complex reconstruction involving vascularized flaps or hardware-dependent constructs [41,42,43]. A series examining immunotherapy and wound complications following flap reconstruction in head and neck cancer patients found an association between checkpoint inhibitor use and wound complications, a finding with plausible relevance to spinal sarcoma reconstruction [43]. As the systemic therapy landscape for spinal sarcomas continues to evolve, the reconstructive team’s engagement in treatment sequencing decisions is essential to optimizing outcomes.

## 4. Surgical Decision-Making as a Biologic, Not Purely Anatomic, Choice

Effective surgical planning in spinal sarcoma integrates oncologic control with a prospective assessment of the biologic environment that reconstruction must navigate. This is not a sequential process (resect first, reconstruct second) but an iterative one in which the anticipated reconstructive strategy should influence the scope of resection, and vice versa.

### 4.1. Staging Frameworks and Their Reconstructive Implications

The Enneking staging system and the Weinstein–Boriani–Biagini (WBB) framework remain the primary tools for surgical planning [9,44]. The WBB clockface model maps transverse and longitudinal tumor extent within and beyond the vertebra, directly informing whether en bloc resection is feasible and anticipating the degree of column destabilization that follows. From a reconstruction standpoint, the WBB map is equally valuable for identifying which vascular pedicles will be preserved or sacrificed, information that determines whether pedicled VBG options will be available.

### 4.2. The Vascular Planning Imperative

Vascular planning in spinal sarcoma is more complex than in most oncologic surgery because the same segmental vessels that supply the spinal cord also supply potential pedicled reconstructive options. Preoperative embolization, while effective in reducing intraoperative blood loss from hypervascular tumors such as osteosarcoma and some chordomas, must be planned in explicit coordination with the reconstructive team to avoid sacrificing the pedicle upon which the VBG depends. This requires preoperative CTA or MRA to map the vascular anatomy of both the tumor and the candidate donor sites, and multidisciplinary discussion before the first incision.

Similarly, in revision settings where prior surgery has disrupted segmental anatomy, the available VBG options may be constrained. Recognizing this preoperatively, rather than discovering it intraoperatively, is the difference between a planned compromise and an unforced error.

### 4.3. Margin Philosophy in the Context of Biologic Heterogeneity

The relationship between margin status and local recurrence varies substantially by histotype. Chordoma and chondrosarcoma are dominated by local failure, and margin-negative resection is the primary determinant of disease-free survival [15,16,17,18]; for these entities, accepting a marginal resection to preserve a particular VBG donor site is likely a poor tradeoff. Ewing sarcoma and osteosarcoma carry systemic metastatic risk that may ultimately be the limiting factor in survival [14,19,20], and the calculus of marginal resection is more nuanced. Notably, a recent pooled analysis of primary spinal sarcomas found that en bloc excision significantly improved overall survival in chondrosarcoma and Ewing sarcoma but did not reach statistical significance for spinal osteosarcoma, suggesting that the survival benefit of aggressive resection may be histotype-dependent and that systemic disease control remains the dominant prognostic factor in spinal osteosarcoma [45]. These distinctions should be explicit in multidisciplinary planning discussions and documented in operative notes. They are the precision-medicine content of surgical decision-making.

### 4.4. Multidisciplinary Coordination and Operative Sequencing

The biologic and treatment-related factors described in Section 3 can only be acted upon if the reconstructive team is engaged before the oncologic treatment plan is finalized, not after. This requires explicit multidisciplinary discussion at the time of radiation planning and systemic therapy selection, so that field design, dose fractionation, chemotherapy cycling, and operative timing can be coordinated prospectively. The practical implications are substantial: pedicled VBG donor vessel preservation requires identifying those vessels before radiation fields are drawn; operative timing relative to chemotherapy nadir requires coordination with the oncologic team weeks in advance; and the decision to use a free versus pedicled VBG may hinge on recipient vessel anatomy that should be characterized preoperatively (Section 4.2) rather than discovered intraoperatively. Documenting the rationale for these sequencing decisions explicitly in the multidisciplinary record creates accountability and provides the oncologic context necessary to interpret postoperative wound behavior should complications arise.

## 5. Established Reconstructive Strategies: A Critical Synthesis

Conventional reconstructive strategies for spinal sarcoma defects have been developed primarily in the context of spinal trauma, degenerative disease, and metastatic tumors. Their application to primary sarcoma, where patients are younger, defects are larger, radiation doses are higher, and survival horizons are longer, reveals important limitations that must be explicitly acknowledged rather than minimized. A systematic review and meta-analysis of 38 studies found a pooled fixation mechanical failure rate of 10% after spinal tumor resection, with tumor level and cage subsidence as significant risk factors [46].

### 5.1. Avascular Grafts: Indications and Limitations

Structural allografts provide immediate mechanical continuity and can be fashioned to match large defects. Their integration depends entirely on creeping substitution, the slow process of host revascularization and remodeling, which is substantially impaired in irradiated tissue [25]. Pseudarthrosis rates approaching 30–40% have been reported in complex spinal applications, with higher rates in previously irradiated beds [26,29]. Non-vascularized autografts (iliac crest, fibula) offer superior osteogenic potential but face the same dependence on host vascularity, with the additional limitations of graft size and donor site morbidity.

These tools are most appropriate for smaller defects in well-vascularized, unirradiated beds, or in patients with limited life expectancy in whom the time horizon for mechanical failure is short. In the precision-medicine context of spinal sarcoma, neither condition is commonly met: patients are often young, and radiation is a standard component of management for several key histotypes.

### 5.2. Metallic Implants and Instrumentation

Titanium cages and expandable prostheses offer robust immediate load-bearing capacity and have become standard components of anterior column reconstruction. Their fundamental limitation, biological inertness, means that long-term stability depends on achieving osseointegration between implant and adjacent vertebra, a process that fails reliably in poorly vascularized environments [47]. Subsidence rates exceeding 28% have been reported for titanium mesh cages, contributing to neurologic deterioration and hardware failure in a proportion of patients [48]. Patient-specific implants and 3D-printed constructs represent an evolution of this approach and are discussed in depth in Section 9.

### 5.3. The Case for Biologic Reconstruction: Summary of Evidence

The evidence base for VBG-based reconstruction in spinal oncology is predominantly composed of small, single-center retrospective case series (Level IV evidence), and this must be stated clearly to avoid evidentiary overstatement. Direct comparative studies of vascularized versus avascular grafts in spinal sarcoma are scarce. A systematic review and pooled analysis from Johns Hopkins pooling 21 studies and 209 patients reported an 89% union rate for VBG-based spinal reconstruction, with an overall complication rate of 42% and reoperation rate of 27% [49]. A 39-patient series from Massachusetts General Hospital using bilateral free vascularized fibula grafts reported union in 76% of evaluable patients at a median follow-up of 50 months [50]. Additional independent evidence from Winters et al. supports the role of free vascularized bone grafts in spinal reconstruction, reporting favorable outcomes in a series of complex spinal defects [51]. The conclusions below should be understood as a synthesis of the best available evidence in a data-sparse field, with the acknowledgment that they are hypothesis-generating rather than definitively established.

With that framing, the pattern across published series is consistent and clinically meaningful: VBGs demonstrate superior arthrodesis rates compared with avascular alternatives in compromised host environments, with pedicled VBG series reporting fusion rates of 78–100% in settings including prior radiation, infection, and revision surgery [50,52,53,54,55,56,57,58,59] (Table 1). The biological mechanism is well-characterized [60], by preserving a viable cell population including osteocytes, osteoblasts, and osteoprogenitor cells, VBGs undergo active primary bone healing rather than the slow secondary revascularization upon which avascular grafts depend.

### 5.4. Spinoplastic Reconstruction: Principles and Framework

The term spinoplastic reconstruction refers to a conceptual framework that combines pedicled VBGs with standard spinal instrumentation to biologically augment fusion across spinal defects, particularly in compromised hosts [55]. The three operational components are: (1) immediate mechanical stability achieved with instrumentation; (2) biologic fusion promoted by contouring and fixing the VBG to bridge the osseous defect; and (3) soft tissue coverage supplemented by local muscle flaps to protect the underlying reconstruction and reduce wound complications.

The pedicled VBG options most commonly employed in spinoplastic reconstruction include the rib (R-VBG, pedicled on intercostal vessels), iliac crest (IC-VBG, pedicled on the iliolumbar artery), clavicle (C-VBG, pedicled on the sternocleidomastoid), occiput (O-VBG, pedicled on the splenius capitis and semispinalis capitis), and scapula (S-VBG, pedicled on the rhomboid and trapezius). Each has distinct arcs of rotation, structural properties, and regional applicability. The precise technical details of each graft, including vascular pedicle anatomy, harvest technique, anastomotic configuration for free variants, and fixation strategies, are described in detail in the source literature and are not reproduced here in the interest of maintaining the precision-medicine framing of this review [56,57,58,61,62,63,64,65,66].

## 6. The Precision-Oriented Reconstructive Ladder

The concept of a reconstructive ladder has been well-established in plastic surgery as a framework for organizing interventions by complexity and biological demand. We propose here an adaptation of this concept specifically for spinal sarcoma reconstruction, explicitly framed as a hypothesis-generating conceptual tool rather than a prescriptive algorithm. No published decision algorithm currently exists for reconstructive strategy selection in spinal sarcoma; existing staging frameworks (WBB, Enneking, NOMS, SINS) address tumor staging, prognosis, and resection approach but not reconstruction [9,67]. Recent adaptations of the reconstructive ladder for extremity sarcoma, including the A-SARC decision score and personalized resection–reconstruction algorithms, demonstrate the feasibility and clinical value of incorporating oncologic variables into reconstructive decision-making [68,69]. Decisions in individual patients will require integration of factors not captured in any ladder, including patient-specific comorbidities, institutional expertise, and patient preference regarding operative risk.

**Figure 2:**
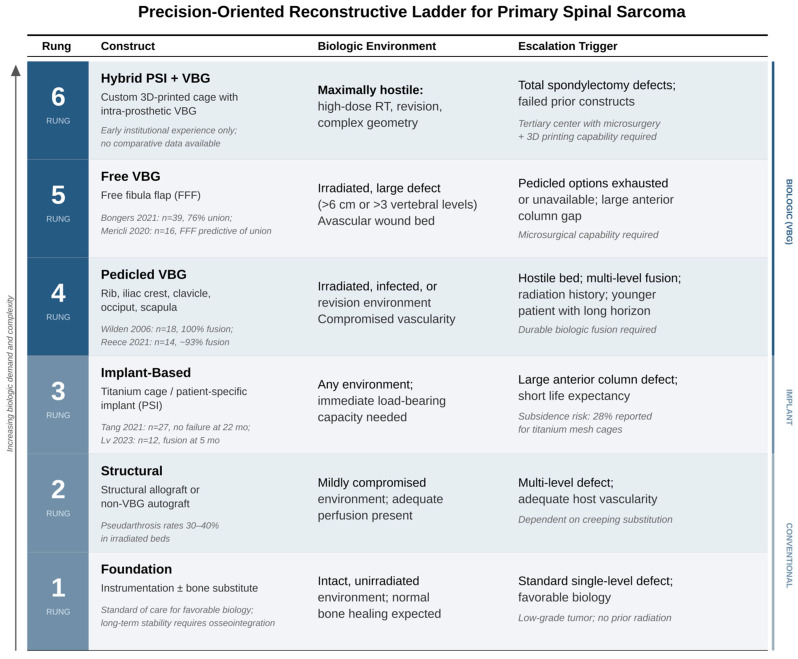
Precision-Oriented Reconstructive Ladder for Spinal STS. Six rungs of increasing biologic demand and complexity, from instrumentation alone (Rung 1) through hybrid PSI-VBG constructs (Rung 6). Escalation is driven by defect magnitude, host biologic compromise, and expected time horizon. The ladder is not strictly hierarchical; Rungs 3 and 4 are frequently combined and Rung 6 represents a qualitatively different approach rather than an escalation of Rung 5.

The precision-oriented reconstructive ladder for spinal sarcoma proceeds from a foundation of instrumentation and structural support through increasingly biologically active reconstructive options, with escalation driven by the magnitude of defect, the degree of host biologic compromise, and the expected time horizon over which the construct must perform. Critically, the ladder is not hierarchical in the sense that higher rungs are always better; they are appropriate for specific clinical contexts and are associated with greater operative complexity, resource requirements, and morbidity (Figure 2).

The decision to escalate is driven by three converging factors assessed preoperatively. First, defect magnitude: defects spanning more than 6 cm or more than 3 vertebral levels generally exceed the capacity of avascular reconstruction and warrant consideration of VBG-based strategies. Second, host biologic compromise: prior radiation at doses exceeding 45 Gy, revision surgery with disrupted segmental vascularity, or active infection each independently increase the likelihood of avascular graft failure and should prompt escalation. Third, expected time horizon: younger patients with favorable oncologic prognosis require constructs capable of decades of biomechanical loading, favoring biologic strategies that achieve true osseous fusion over implant-only approaches vulnerable to long-term fatigue failure. When two or more of these factors converge, the indication for biologic reconstruction is strongest. Table 2 illustrates how these factors interact in representative clinical scenarios.

Several aspects of this ladder merit explicit comment. First, the ladder is not strictly sequential; PSIs (Rung 3) and pedicled VBGs (Rung 4) are frequently used in combination, with the PSI providing immediate stability while the VBG provides long-term biologic fusion. Second, the hybrid PSI-VBG construct (Rung 6) is not simply an escalation of Rung 5; it represents a qualitatively different approach that combines the strengths of implant-based and biologic strategies. Third, and most importantly, the clinical context that triggers escalation, particularly the presence of prior radiation, revision surgery, or large multi-column defects, should be identified proactively in preoperative planning, not recognized intraoperatively when options may be constrained. An AOSpine Knowledge Forum Tumor systematic review with Delphi consensus concluded that structural bone graft with or without cage should be considered for defects spanning more than two vertebral bodies, and that planned postoperative radiation should explicitly guide surgical fusion strategy [67].

## 7. Regional Considerations: Tailoring Strategy to Anatomy

**Figure 3:**
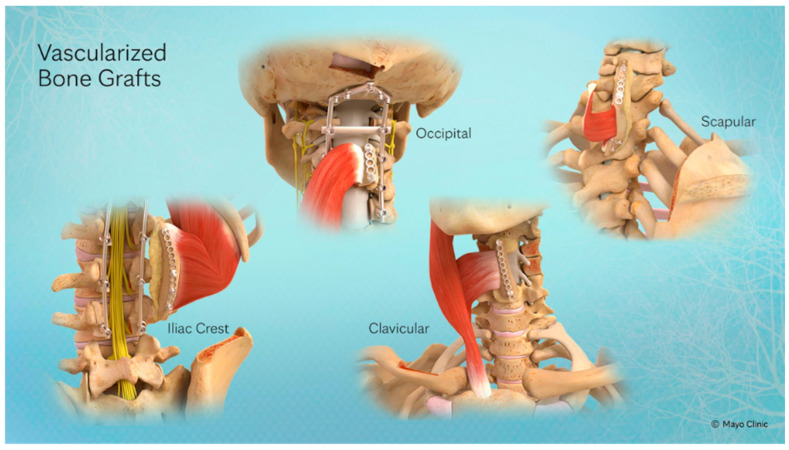
Vascularized bone graft options for spinal reconstruction. Anatomical illustration of four pedicled VBG options used in spinoplastic reconstruction: occipital (pedicled on splenius capitis and semispinalis capitis), clavicular (pedicled on sternocleidomastoid), scapular (pedicled on rhomboid and trapezius), and iliac crest (pedicled on iliolumbar artery). Instrumentation and soft tissue pedicle relationships are shown. Illustration courtesy of Mayo Clinic.

The precision-oriented reconstructive framework must be applied within the anatomic constraints and biomechanical demands specific to each spinal region. The following regional summaries are intended to highlight the precision-medicine content of regional decision-making, not to provide comprehensive technical descriptions (Table 3, Figure 3).

### 7.1. Cervical Spine

The cervical spine’s high mobility and proximity to critical neurovascular, respiratory, and digestive structures make reconstruction uniquely demanding. Even limited resections may destabilize the column, and the compact anatomy limits the reconstructive toolkit. For most sarcoma resections requiring multilevel anterior column reconstruction, the free vascularized fibula graft is the workhorse option. Its cortical strut provides structural support while its preserved vascularity enables reliable fusion in the irradiated fields common after cervical sarcoma treatment [70,71]. The pedicled clavicular VBG is an effective option for shorter segments. Cervical reconstruction is also where the precision-medicine principle of vascular planning is most critical: the superior thyroid and transverse cervical arteries serve as recipient vessels for free fibula anastomosis and must be identified and preserved during tumor resection.

### 7.2. Thoracic Spine

The thoracic spine is the most common location for primary spinal sarcomas, particularly Ewing sarcoma and osteosarcoma. The rib cage provides supplemental stability that is absent elsewhere in the spine, but en bloc resections still create multi-column defects that require combined anterior and posterior reconstruction [52,65]. The pedicled rib VBG is the primary biologic option for thoracic reconstruction, with its long arc of rotation based on posterior intercostal vessels allowing posterior onlay grafting across multiple levels. Critically, the rib VBG’s structural strength is limited, making it best suited as a biologic complement to a primary mechanical reconstruction rather than a sole load-bearing element. Protecting the intercostal pedicle from posterior instrumentation requires deliberate planning and intraoperative attention.

### 7.3. Lumbosacral Spine

Reconstruction at the lumbopelvic junction presents some of the most formidable challenges in spinal oncology. The transition from mobile lumbar spine to weight-bearing sacrum creates complex biomechanics, and sacrectomy defects, common in chordoma management, require reconstruction strategies that restore load transfer to the pelvis while maintaining fusion in an anatomic region subject to high cyclical stresses [72,73,74]. The pedicled iliac crest VBG, based on the iliolumbar artery, is the primary biologic option; it provides a robust source of corticocancellous bone that can be rotated into lumbar or sacral defects without microsurgical anastomosis. Stable lumbopelvic fixation using iliac or S2AI screws is not optional; it is the mechanical foundation without which no biologic strategy will succeed. For very large defects or when the iliac crest is unavailable due to tumor involvement or prior harvest, free fibula grafting with anastomosis to iliolumbar or superior gluteal vessels remains an option at centers with microsurgical capability.

## 8. Perioperative Management: Protecting the Reconstruction

The complexity of spinoplastic reconstruction extends into the perioperative period. Meticulous perioperative management is as important as the surgical strategy itself in determining ultimate outcomes.

### 8.1. Soft Tissue Coverage

Soft tissue coverage deserves particular emphasis in spinal sarcoma, where prior radiation substantially increases the risk of wound dehiscence and deep infection over exposed instrumentation and grafts. Local muscle flaps (paraspinous, gluteus maximus, and trapezius) remain the workhorses for posterior spinal coverage. For high-risk closures involving large sacral and lumbosacral defects, particularly after total sacrectomy, the selection of soft tissue reconstruction strategy carries meaningful consequences for morbidity. Falade et al. reported a 28-patient UCSF series of soft tissue reconstruction after sacral neoplasm resection for chordoma and chondrosarcoma, with a mean follow-up of 4 years; the major wound complication rate was 42.9%, VRAM flaps were associated with a statistically higher number of reoperations and longer hospital stay than gluteal-based flaps, and, notably, radiation therapy was not significantly associated with higher overall wound complication rates in this cohort [75,76]. The last finding is clinically meaningful: it suggests that flap selection and execution, rather than radiation history alone, may be the more modifiable determinant of wound outcome in this population. For large defects after total sacrectomy the preferred reconstruction was a combination approach using gluteal-based muscle flaps, while local fasciocutaneous flaps were favored for small and medium-sized defects.

Pedicled omental flaps have emerged as a valuable adjunct for the highest-risk closures. Massaad et al. reported a retrospective series of 34 patients, including 28 chordomas, 4 chondrosarcomas, 1 Ewing sarcoma, and 1 giant cell tumor, of whom 27 had received prior radiation, with a median follow-up of 24 months (range 0 to 132 months); the omentum was used primarily for soft tissue coverage in 59% of cases and was reported as effective for high-risk closures in this heavily irradiated, histologically diverse cohort [77]. The omentum’s rich vascularity, capacity to obliterate dead space, and intrinsic resistance to infection make it particularly well-suited to the hostile wound environments common in sarcoma surgery.

### 8.2. Postoperative Monitoring and Recovery

For free vascularized grafts, postoperative monitoring is essential during the critical 48–72 h window of maximal thrombosis risk. This typically involves a combination of clinical assessment and implantable or surface Doppler monitoring. While there is no universal consensus on perioperative anticoagulation, many centers employ aspirin or low-dose heparin regimens to reduce thrombosis risk without substantially increasing hemorrhagic complications [78].

Enhanced Recovery After Surgery (ERAS) principles adapted for complex spinal oncologic procedures should include preoperative nutritional optimization, particularly relevant in sarcoma patients who may be nutritionally depleted from prior systemic therapy, as well as goal-directed fluid management, and early mobilization protocols. Preoperative albumin and prealbumin assessment, with nutritional intervention where deficient, can meaningfully reduce wound complication rates in this population [79].

### 8.3. Donor Site Management

Donor site management deserves equal attention. Iliac crest harvest is associated with chronic pain in a proportion of patients [53,54]; rib harvest can produce chest wall discomfort and contour deformity [65]; fibula harvest, while well tolerated in most patients, sacrifices one of the leg’s primary vascular axes and requires careful patient counseling and postoperative rehabilitation [50,59]. These trade-offs should be explicitly discussed in preoperative informed consent and documented in the operative record.

## 9. Integration with Emerging Technologies: The Hybrid Frontier

The convergence of biologic reconstruction with advanced implant technology represents the most hypothesis-generating area in the field. At present, the available evidence is limited to small institutional experiences and conceptual reports; the section below is explicitly framed as a discussion of emerging directions rather than established practice.

### 9.1. Patient-Specific 3D-Printed Implants: Mechanical Precision Without Biologic Reliability

Patient-specific implants (PSIs) represent a meaningful advance in mechanical design, offering precise anatomical matching and optimized load distribution for complex, asymmetric post-resection defects [80]. For patients with limited life expectancy or in regions where VBG options are technically unavailable, PSIs may represent the optimal primary strategy. Tang et al. reported preliminary results of a 3D-printed modular vertebral prosthesis for anterior column reconstruction after multilevel thoracolumbar total en bloc spondylectomy in 27 patients with diagnoses spanning chondrosarcoma, osteosarcoma, Ewing sarcoma, malignant peripheral nerve sheath tumor, and undifferentiated high-grade pleomorphic sarcoma; at mean follow-up of 22 months, 26 patients with minimum one-year follow-up showed no evidence of internal fixation failure or prosthesis dislocation, and at latest follow-up, 19 of 23 surviving patients could walk independently [81]. Lv et al. evaluated a novel biomechanically validated custom 3D-printed sacral prosthesis in 12 patients undergoing total en bloc sacrectomy, including 7 chordomas, 3 osteosarcomas, 1 chondrosarcoma, and 1 undifferentiated pleomorphic sarcoma, reporting satisfactory osseointegration in all patients with a mean fusion time of 5 months and improvement in quality of life and pain scores at mean follow-up of 38.5 months; notably, only one patient received adjuvant radiotherapy, limiting conclusions about performance in irradiated fields [82]. Lador et al. further illustrated the utility of 3D-printed anatomical models and custom implants in improving preoperative planning and intraoperative decision-making for complex spinal oncologic cases [83,84,85]. A recent systematic review and meta-analysis comparing 3D-printed artificial vertebral bodies to conventional titanium mesh cages across 9 studies and 374 patients found that 3D-printed constructs were superior in reducing operation time, blood loss, postoperative pain, vertebral body subsidence, and early complications after total en bloc spondylectomy [86]. However, follow-up in all published series is short relative to the expected survival horizon of younger sarcoma patients, and radiation exposure in most cohorts is minimal, limiting conclusions about PSI performance in the hostile biologic environments that are precisely the most common drivers of reconstructive complexity in spinal sarcoma. No study has specifically isolated outcomes in irradiated patients receiving 3D-printed spinal implants, representing a notable evidence gap.

### 9.2. Hybrid PSI–VBG Constructs: Rationale and Early Experience

The hybrid construct concept, combining the mechanical precision of a custom PSI with the biologic reliability of a VBG, represents a theoretical resolution of the tension between immediate stability and long-term fusion. In the hybrid approach, a custom 3D-printed vertebral body or cage is designed with a central channel or portal that accepts a pedicled or free VBG, most commonly a fibula or iliac crest graft. The VBG functions as a living ‘fusion engine’ within the implant, creating a solid arthrodesis that integrates the construct and reduces the hardware’s vulnerability to long-term fatigue failure [47].

This approach remains exploratory. Published experience consists of small, single-institution series, and no comparative data exist. The resource requirements are substantial, requiring simultaneous expertise in oncologic spine surgery, microsurgery, and 3D implant design and manufacturing, and are currently available only at a limited number of tertiary academic centers. Hybrid PSI-VBG constructs should therefore be considered a potential future direction suited to highly selected patients at appropriately resourced institutions, rather than a generalizable standard approach. We present it here because it exemplifies the hypothesis-generating innovations most needed in this field, and because understanding its logic clarifies the broader principle that biologic and mechanical strategies are most powerful in combination.

### 9.3. Bone Morphogenetic Proteins: A Contested Adjunct

Bone morphogenetic proteins (BMPs), particularly rhBMP-2, have been used as biologic adjuncts to promote fusion in complex spinal reconstruction. Their use in oncologic spine surgery is complicated by concerns regarding pro-proliferative and pro-angiogenic effects of BMP signaling, which raise theoretical concerns about tumor promotion in the operative field [87]. Published data are conflicting: several retrospective analyses in degenerative populations have reported small increases in new cancer diagnoses after rhBMP-2 exposure, while multiple large registry studies and independent re-analyses have found no clear malignancy risk increase [88,89]. Evidence specific to patients with active or recently treated spinal sarcoma is essentially absent. Until prospective data are available in oncologic populations, most spinal sarcoma programs have appropriately avoided BMP in or near the tumor bed in favor of autograft, allograft, or VBG-based biologic strategies.

### 9.4. Preoperative Vascular Mapping and Intraoperative Technology

Advanced preoperative CTA and MRA allow precise characterization of the vascular anatomy relevant to both tumor resection and VBG harvest, enabling the multidisciplinary team to identify recipient vessel options, anticipate pedicle conflicts with instrumentation, and plan anastomotic configurations before the first incision [83]. This planning capacity is particularly valuable in revision cases where prior surgery has altered normal anatomy. Intraoperative navigation and robotic assistance improve the accuracy of instrumentation placement and tumor resection, with potential benefits in preserving vascular pedicles and reducing the margin of error in complex reconstructions. Patient-specific surgical guides, produced in parallel with PSI manufacturing, can further reduce operative time and technical variability [80,85].

## 10. Reconstruction as an Enabler of Oncologic Innovation

A central thesis of this review is that reconstruction in spinal sarcoma should be understood not merely as repair of defects created by oncologic surgery, but as an active component of the oncologic treatment strategy. The quality and durability of reconstruction directly determines the degree to which patients can tolerate subsequent systemic therapy, tolerate revision surgery when needed, and maintain the neurologic and functional status necessary to participate in emerging investigational treatments.

### 10.1. Enabling Systemic Therapy Delivery

The relationship between reconstruction quality and systemic therapy delivery is underappreciated in the spinal sarcoma literature. Wound complications following avascular reconstruction in irradiated beds, including dehiscence, infection, and hardware exposure, can delay or preclude adjuvant chemotherapy, create systemic inflammatory burdens that interact with immunotherapy responses, and necessitate revision surgeries that compete with oncologic follow-up [33,35]. By providing a more reliably vascularized wound environment, VBG-based reconstruction has the potential to reduce the wound complication rate and thereby preserve the patient’s access to the full spectrum of systemic treatment.

The hypothesis that reconstruction quality affects systemic therapy tolerance has not been prospectively tested in spinal sarcoma, and we identify it as a priority for future investigation. The relevant endpoints would include time to initiation of adjuvant therapy, number of therapy cycles delayed or omitted due to wound complications, and survival outcomes stratified by reconstructive approach.

### 10.2. Radiation Timing and Reconstructive Strategy

The interaction between radiation therapy and reconstructive biology is bidirectional. As discussed in Section 3, prior radiation compromises host vascularity and impairs avascular graft incorporation. But the timing of reconstruction relative to radiation also matters in the reverse direction; reconstruction that creates a vascularized, well-covered wound environment may expand the safe delivery window for adjuvant radiation, particularly in cases where wound healing concerns would otherwise mandate dose reduction or field modification.

This is relevant to emerging radiation strategies including intensity-modulated radiation therapy (IMRT) and proton beam therapy, which allow more precise dose delivery to the tumor bed while sparing adjacent critical structures [90]. The potential synergy between precision radiation delivery and precision reconstruction, each designed to maximize oncologic efficacy while minimizing host morbidity, represents a hypothesis-generating framework for future translational investigation.

### 10.3. Future Gene and Cell Therapy Delivery Paradigms

Emerging innovations in sarcoma management include regional delivery of gene therapy vectors, adoptive cell therapy, and checkpoint inhibitor payloads to the tumor bed. These approaches are theoretically enhanced by a well-vascularized local environment, which VBG-based reconstruction provides. The hypothesis that biologic reconstruction might serve as a delivery platform for regional biotherapy, rather than merely a structural repair, has not been explored in spinal sarcoma but merits consideration as the field matures.

## 11. Limitations, Unmet Needs, and Hypothesis-Generating Frameworks for Future Investigation

The field of spinal sarcoma reconstruction is constrained by the same limitation that affects all rare-disease surgery: the evidence base is dominated by small, retrospective, single-institution series that cannot support the strong comparative claims sometimes made on their behalf. The following section identifies the specific gaps where targeted investigation would most meaningfully advance the field.

### 11.1. Standardized Outcome Definitions

The absence of standardized outcome definitions is the most tractable barrier to progress. Fusion, as an endpoint, is currently defined inconsistently across studies. Some use plain radiography, others CT; some define union at 6 months, others at 1 year. The distinction between ‘probable union’ and ‘definite union’ varies by author. A consensus definition framework, specifying imaging modality, timing windows, and criteria for union, pseudarthrosis, and hardware failure, is a prerequisite for any meaningful comparative analysis. Similarly, standardized complication reporting (distinguishing wound dehiscence, infection, hardware failure, local recurrence, and reoperation as distinct endpoints with defined windows) would enable meta-analytic synthesis that is currently impossible.

### 11.2. Prospective Registries

Given the rarity of spinal sarcoma, single-institution prospective trials are unlikely to achieve the sample sizes necessary for definitive comparative conclusions. Multi-institutional prospective registries, organized around the standardized definitions proposed above, represent the most realistic path to Level II or III evidence. Such registries would be most valuable if they captured not only reconstructive outcomes but also oncologic outcomes (local recurrence, distant failure, survival) and patient-reported outcomes (functional status, pain, quality of life), enabling analysis of whether reconstruction quality affects the delivery of systemic therapy and long-term survival.

### 11.3. Histotype-Stratified Analysis

The heterogeneity of spinal sarcoma, encompassing entities with fundamentally different biologic behaviors, systemic therapy sensitivities, and recurrence patterns, means that pooled analyses across histotypes have limited interpretive value. Future studies should be designed and reported with histotype stratification as a primary analytical framework, enabling the identification of which reconstructive strategies are most appropriate for which tumor types. This is particularly relevant for the question of VBG timing relative to radiation and systemic therapy, where the optimal sequence may differ substantially between, for example, chemosensitive Ewing sarcoma and chemoresistant chordoma.

### 11.4. Decision Algorithms and Risk Stratification

The precision-oriented reconstructive ladder proposed in Section 6 is a conceptual framework, not a validated clinical decision algorithm. Prospective validation of a decision algorithm, one that assigns reconstructive strategy based on preoperative assessment of defect magnitude, host biologic environment, and expected time horizon, would represent a meaningful advance. Such an algorithm could be developed and validated across the multi-institutional registry proposed above. The specific decision nodes with the highest current uncertainty include: (1) the threshold radiation dose beyond which pedicled VBG becomes the default rather than the optional strategy; (2) the defect size and column involvement threshold that favors free over pedicled VBG; and (3) the criteria for hybrid PSI-VBG versus PSI-alone.

### 11.5. Cost-Effectiveness and Generalizability

Complex VBG-based reconstruction and hybrid constructs require substantial institutional investment: experienced combined surgical teams (spine and microsurgery), ICU monitoring infrastructure, 3D printing capability, and the operative time and resource commitment of prolonged combined procedures. These requirements limit the generalizability of outcomes reported from high-volume tertiary centers to the broader population of patients with spinal sarcoma. Understanding the cost-effectiveness of biologic reconstruction strategies and identifying the thresholds of patient complexity beyond which the investment is justified are essential for equitable access. This analysis does not currently exist in the spinal sarcoma literature and is identified as a priority.

## 12. Conclusions

Reconstruction following the resection of primary spinal sarcomas is among the most technically and biologically demanding challenges in musculoskeletal oncology. This review has argued for a precision-medicine framework that anchors reconstructive decision-making in tumor biology, host environment, and expected time horizon rather than in anatomic convention or institutional preference. The central empirical finding, that VBG-based spinoplastic reconstruction achieves reliable fusion in compromised host environments where avascular constructs fail, is supported by a consistent pattern across published series and by independent pooled analyses reporting 89% union rates [49], with the explicit caveat that the evidence base is comprised predominantly of small, retrospective series and that comparative conclusions must be considered hypothesis-generating rather than definitive. The precision-oriented reconstructive ladder proposed here is a conceptual framework, not a validated clinical algorithm, and it requires prospective, multi-institutional validation before adoption as a clinical standard.

Filling the evidence gaps identified in this review, through standardized outcome definitions, multi-institutional prospective registries, histotype-stratified analysis, and comparative studies of VBG-based versus conventional reconstruction in matched populations, is the work of the field’s next decade. Until such data are available, the integration of reconstruction as a proactive, biologically informed component of multidisciplinary oncologic planning, rather than a reactive surgical afterthought, represents the most actionable contribution of this framework to current practice.

### Limitations

This review has several important limitations that must be stated explicitly. The evidence base for VBG-based reconstruction in spinal sarcoma is comprised almost entirely of small, single-center retrospective case series (Level IV evidence). No randomized controlled trials or prospective comparative studies exist. The conclusions of this review should therefore be understood as a synthesis of the best available evidence in a data-sparse field, with the strength of any comparative claims limited accordingly.

Definitions of fusion, imaging modalities, and minimum follow-up intervals varied substantially among included studies, making direct comparison of reported fusion rates difficult. Patient populations in published series are heterogeneous with respect to tumor histology, radiation history, and indication for VBG reconstruction, further limiting pooled interpretation. The hybrid PSI-VBG framework presented in Section 9.2 is based on early institutional experience without comparative data.

Finally, we acknowledge that this review originates from a center with particular expertise and interest in VBG-based spinoplastic reconstruction. While we have made explicit efforts to present evidence in a balanced and guarded manner, readers should weigh our conclusions in the context of the broader literature and the limitations of the evidence base.

## Figures and Tables

**Table 1:** Consolidated summary of key clinical series on VBGs for spinal reconstruction.

Author/Year	*n*	Pathology	VBG Type	Region	Fusion Outcome	Notes
Wilden et al., 2006 [52]	18	Malignancy, infection, trauma	Pedicled rib (R-VBG)	Thoracic	100% reported fusion rate	6 prior infections; 1 spinal metastasis
Reece et al., 2021 [53]	14	Failed fusion/pseudoarthrosis	Pedicled iliac crest (IC-VBG)	Lumbar	93% reported fusion rate	All revision; compromised wound beds
Mericli et al., 2020 [59]	40	Oncologic vertebrectomy	Free fibula flap (FFF)	Mixed	High (bony union predictive)	FFF significantly predicted bony union; 40-patient cohort (16 FFF vs. 24 controls)
Bongers et al., 2021 [50]	39	Tumor Resection	Free fibula flap (FFF)	Mobile spine	No nonunion reported	Multi-institutional; surgical technique described

**Table 2:** Representative Clinical Scenarios Illustrating Precision-Oriented Reconstructive Decision-Making. These hypothetical vignettes demonstrate how specific combinations of tumor biology, host environment, anatomic level, and expected survival horizon converge to guide ladder rung selection. Each scenario represents a composite clinical picture informed by published evidence; individual patient decisions require integration of additional factors not captured in any framework.

Scenario	Patient Profile	Key Biologic Factors	Recommended Rung	Rationale	Caveats
A	55 y/o, low-grade chondrosarcoma, single thoracic vertebra, no radiation, en bloc with clear margins	Favorable biology; intact vascularity; standard defect	Rung 1–2 (Foundation/Structural)	Uncompromised host; small defect; long survival horizon but favorable environment for avascular fusion	Monitor for late adjuvant RT need; if radiation planned, escalate to Rung 4
B	32 y/o, Ewing sarcoma, T8–T10, neoadjuvant chemoradiation (54 Gy), multilevel TES planned	Irradiated bed (>45 Gy); large defect (3 levels); young patient; long survival horizon	Rung 4–5 (Pedicled or Free VBG)	Avascular graft failure risk high in irradiated field; young patient requires durable biologic fusion over decades	Rib VBG if intercostal pedicle preserved; escalate to free fibula if pedicled options compromised by RT field
C	48 y/o, recurrent sacral chordoma, prior proton beam (74 Gy), prior instrumentation, total sacrectomy	Maximally hostile: high-dose RT, revision setting, complex geometry	Rung 5–6 (Free VBG or Hybrid PSI + VBG)	Pedicled options likely exhausted; recipient vessels may be compromised; PSI provides immediate stability while VBG provides biologic fusion	Requires tertiary center with microsurgery + 3D printing; preoperative CTA essential for recipient vessel mapping
D	70 y/o, high-grade UPS, L2, limited life expectancy, adjuvant RT planned	Short survival horizon; RT planned but limited biologic investment warranted	Rung 3 (PSI/Implant-Based)	Immediate stability priority; limited survival horizon makes long-term fusion less critical than in younger patients	If patient exceeds expected survival, late hardware failure may require revision with biologic escalation

**Table 3:** Precision-oriented regional reconstructive strategies for spinal sarcomas. Primary and alternate VBG options by region, with the key biomechanical consideration and clinical indication that should trigger VBG selection over avascular alternatives.

Region	Primary VBG Option	Alternative Option	Key Biomechanical Considerations	Precision-Oriented Indication
Cervical	Free fibula (FFF), anterior multilevel	Pedicled clavicle (C-VBG), occipital (O-VBG), short segments	High mobility; circumferential stability critical	Radiation history; multilevel corpectomy; biologically aggressive histology
Thoracic	Pedicled rib (R-VBG), posterior onlay	Pedicled scapula (S-VBG), pedicled spinous process (SP-VBG)	Rib = biologic onlay; must supplement cage for load	Post-radiation bed; multilevel posterior fusion augmentation
Lumbosacral	Pedicled iliac crest (IC-VBG)	Free fibula if iliac unavailable	Cantilever forces; S2AI fixation advised	Sacrectomy defects; prior radiation; revision pseudoarthrosis

## Data Availability

No new data were created or analyzed in this study.
